# Interfacility Transfers from the Emergency Department for Non-contracted Insurance Status Disproportionately Affect Minority Patients

**DOI:** 10.5811/westjem.47200

**Published:** 2025-11-18

**Authors:** Andrew Holzman, Malik Aaron, Krish Nayar, William Rankin, Melissa Tapia, Douglas Rappaport

**Affiliations:** * Alix School of Medicine, Mayo Clinic, Phoenix, Arizona; † Mayo Clinic Hospital, Rochester, Department of Care Management, Phoenix, Arizona; ‡ Mayo Clinic Hospital, Department of Emergency Medicine, Phoenix, Arizona

## Abstract

**Introduction:**

Transfers between emergency departments (ED) can have an important impact on patient care and experience. We examined interfacility transfers from an academic ED due to insurance status to determine whether they disproportionately affected minority demographics.

**Objective:**

Our objective was to determine whether interfacility transfers for non-contracted insurance status disproportionately affected minority patients in our hospital ED.

**Methods:**

We extracted data from the hospital’s electronic health record system. Records for patients who underwent facility transfer were reviewed to determine which transfers were due to insurance contracting status. We compared the number of patients transferred for insurance incompatibility with the number admitted to the same hospital as initially seen in the ED, either to observation or inpatient status, for groups with socioeconomic minority status including Hispanic, Hispanic non-White, Black, Native American, and non-English speaking.

**Results:**

We identified 2,031 total interfacility transfers. Of these, 735 (36.2%) met inclusion criteria, and 49.7 % (366/735) of these transfers were due to insurance incompatibility. The total transfer rate for all patients was .93% (366/39,299). Increased transfer rates due to insurance incompatibility were observed for all minority demographics queried. The most severe disparity in effect size was for non-English speakers (2.06% compared to 0.90% for English-speakers; 2.32 odds ratio [OR], P < .001). Patients with Hispanic ethnicity experience insurance transfer in 1.31% of cases compared to 0.87% for non-Hispanic whites (OR 1.52, P < .001). The insurance transfer rate for all non-White patients was elevated at 1.11%, but this did not rise to the level of statistical significance (OR 1.28, P = .06).

**Conclusion:**

In our single-center ED study, minority patient populations were disproportionately impacted by interfacility transfers for non-contracted insurance status. We found increased transfer rates due to insurance incompatibility for all minority demographics queried. The most severe disparity was found for non-English speakers and patients with Hispanic ethnicity.

## INTRODUCTION

In 1986, the US Congress passed the Emergency Medical Treatment and Active Labor Act (EMTALA).^1^. Its entry into law came after numerous reports in the popular press and medical literature describing the practice of patient “dumping,” whereby patients were transferred between facilities for inability to pay before stabilization of their medical condition.^1^ In 1984, one study of dumping practices found that patients subject to these transfers were more likely to be minorities.^2^

The EMTALA was more successful than previously existing measures in ensuring adequate emergency care before transfer, broadly requiring that facilities must medically screen patients for an emergency medical condition and stabilize that condition to the extent a facility’s capability allows without regard to a patient’s ability to pay.^1^ Despite EMTALA’s success in changing emergency department (ED). practice, evidence remains that uninsured patients are more likely to experience inter-hospital transfer from the ED with some diagnoses.^3^

Even in the setting of EMTALA, inter-hospital transfer for insurance status is possible once a condition has been stabilized in the emergent setting. These transfers may still have an impact on patient care, although this potential impact has not been well evaluated by the existing literature. One study examined transfers using data from both a Centers for Medicare & Medicaid Services database and 2013 data from the American Hospital Association, finding that inter-hospital transfer was associated with higher cost, longer overall length of stay, lower odds of discharge to home, and higher risk of 3- and 30-day mortality in certain conditions.^4^ This study was not specific to ED transfers for insurance status and may not be representative of the impact on patient care seen in patients transferred following stabilization of their emergency medical condition.

As part of the 2021 Consolidated Appropriations Act passed by Congress, the No Suprises Act (NSA), was intended to limit the practice of “balance billing” patients for out-of-network care. The act requires facilities to bill at median in-network rates until a patient can consent to safe transfer (which may go beyond the point of stabilization under EMTALA).^5^ The law. sought to limit the cost to patients for out-of-network emergency care.

In its litigation regarding implementation of the NSA, the American College of Emergency Physicians (ACEP) highlighted the effect of insurer negotiating power on the emergency care reimbursement landscape.^6^ A 2015 ACEP poll found that one-third of emergency physicians had considered leaving medicine due to decreases in reimbursement from insurers.^6^ Some advocacy groups have argued, therefore, that the responsibility to avoid exorbitant out-of-network costs to patients rests both with hospitals and with insurers, who can expand their network of coverage by increasing contracted reimbursement rates.^7^ Although no specific data exist to suggest that the NSA has resulted in increased interfacility transfer rates for out-of-network patients, one potential unintended consequence of this legislation is that it financially disincentivizes EDs from managing out-of-network patients beyond the initial medical stabilization required by EMTALA.

**Population Health Research Capsule**

What do we already know about this issue?

*Inter-hospital transfers are associated with higher costs, increased length of stay, and higher 3- and 30-day mortality in certain conditions.*

What was the research question?

*Do emergency department transfers for non-contracted insurance status disproportionately impact minority populations?*

What was the major finding of the study?

*Non-contracted insurance transfers for non[1]English speakers occur more often compared to English speakers (OR 2.32, P < .001) and patients with Hispanic ethnicity compared with non-Hispanic Whites (OR 1.52, P < .001).*

How does this improve p-opulation health?

*This study sheds light on a systemic barrier that minority populations face within our healthcare system and the ethics of transfers for non-contracted insurance status.*

In this study we aimed to highlight the potential importance of social equity in discussions and decisions concerning contracted status and hospital provision of emergency care in the post-EMTALA and No Suprises Act setting.

## METHODS

We extracted data from the hospital’s electronic health record (EHR) (Epic Systems Corporation, Verona, WI) and analyzed the data using Microsoft Excel (Microsoft Corporation, Redmond, WA). The study period covered ED visits from January 1, 2021–December 31, 2023, and records for all patients who presented to the ED during the study period were queried. We included records only if the final disposition was admission to an in-facility floor, the in-facility observations unit, or a facility transfer.

Records for patients who underwent facility transfer were reviewed to determine which transfers were due to insurance contracting status. Abstraction of records was performed by live chart review with each chart reviewed by one reviewer. In designing the chart review, reference was made to universal standards of quality as described by Worster et al, 2005.^8^ Specifically, each abstractor was trained before data collection. Although reviewers were not blinded to the purpose of the study, variables were operationalized objectively. For patients in which the chart made explicit reference to insurance status as a reason for transfer those patients were coded “insurance transfers.” Where there was no overt reference to insurance status (eg, where “patient preference” alone was cited) the chart was coded as a non-insurance transfer. Interobserver reliability was, therefore, not tested due to the objectivity of the review criteria, although quality control was performed by random sampling, with the first author reviewing a selection of each of the other reviewer’s charts to ensure established objective criteria had been followed. We excluded patients transferred to access care not provided at our facility’s hospital. To expedite review, all pediatric patients were excluded given that our facility does not provide pediatric inpatient services, and we also excluded any patient whose principal payor was in-network.

We compared the number of patients transferred for insurance incompatibility with the number admitted either to observation or inpatient status at our hospital for groups with socioeconomic minority status. Race was extracted from the EHR, where it was coded according to inputs obtained from survey data at the time of registration upon presentation to the ED. Race was self-reported by patients on these surveys. We allocated survey field values for race to assigned racial categories for statistical analysis, with values including two racial groups counted twice, except that patients ascribed both “White” and another race were counted in the non-White racial group.

A total of 2,031 records of interfacility transfers and 60,235 records of admissions were identified. Of these, we selected 735 records of interfacility transfer for manual review after the initial screening discussed above. Specifically, we excluded those patients who were transferred for care not available at our facility (pediatrics, trauma, obstetrics) as these transfers were, by definition, unrelated to insurance status.

## RESULTS

We identified 336 transfers due to insurance incompatibility. The total transfer rate for all patients was .93% (366/39,299). Significantly increased transfer rates due to insurance incompatibility were observed for Hispanic patients (1.31% of all patients either admitted or transferred compared to 0.87% for non-Hispanic Whites; 1.52 odds ratio (OR), *P* < .001) and non-English speakers (2.06% compared to 0.90% for English-speakers; 2.32 OR, *P* < .001). The absolute number of patients who experienced transfers due to insurance in the groups of Black (24), American Indian (4) and Asian (14) patients was low. Data for these groups were analyzed in aggregate and did not rise to the level of significance (1.11% of all patients either admitted or transferred compared to 0.87 for non-Hispanic Whites; 1.28 OR = .06). For the combined group of non-White, Hispanic, or non-English speaking patients the rate of transfers due to insurance was 1.30% (compared to 0.84% for White, non-Hispanic English speakers; 1.55 OR, *P* < .001). Among patients transferred for non-contracted insurance status, the most common insurance type was Medicare Advantage plans, with 194 (53%) patients transferred.

## DISCUSSION

Each minority group highlighted in our analysis had a higher rate of insurance transfers than any of the reference non-minority groups. Hispanic and non-Hispanic ethnicities were considered, while race was categorized into groups including White, Asian/Pacific Islander, Black, and Native American. Our dataset would have enabled comparison of transfer rates in each of these individual racial groups to the reference group, but this would have required significant time spent aligning the manual coding of race in inpatient records with categories used in this study. Although statistical significance may have been achieved with respect to one of the subgroup elements, we analyzed the non-White group in aggregate; and since we did not find a significant result, we did not pursue analysis that could have drawn conclusions regarding each of these subgroups.[Table t1-wjem-26-1696][Table t2-wjem-26-1696][Table t3-wjem-26-1696]

Future analysis could use a methodological approach that would make individual subgroup analysis more technically feasible for the size of the study to make further claims in this regard. In addition, the number of patients with non-White race seen in our ED is low. Consequently, the number of patients in these racial categories experiencing insurance transfer was also low in absolute terms. Due to the very low absolute numbers of these patients seen, a healthcare facility serving larger populations of these patients might be better suited to draw granular conclusions with respect to each individual racial category.

For each group other than the combined group of non-White race patients, the odds ratio to the relevant non-minority reference group was statistically significant to *P* < .05. In particular, non-English speaking patients appeared to be significantly burdened by interfacility insurance transfers, with a rate just over 2% compared to 0.9% for English-speaking patients. This is of relevance given the other barriers these patients face in the healthcare system generally.^9^

At our hospital, most insurance transfers (93.7%) were of Medicare and Medicaid patients. Patients with Medicare Advantage plans experienced a major impact. We did not specifically analyze insurance plan type as a risk factor for insurance transfer. This is because an assumption of our study is that the only factor involved in the decision to make an insurance transfer when inpatient treatment is appropriate is whether a patient’s insurance is a contracted type for inpatient admission, and this will be consistent across all patients with a given insurance type. The high percentage of patients with these insurance types in the transferred group, therefore, reflects the non-contracted status of these insurances. Acceptance of Medicaid is voluntary for hospitals, although refusal of Medicaid patients has been a source of debate in medical ethics.^10^

Patients experiencing rejection from private practices due to Medicaid status are more likely to defer care or use hospital EDs to access physicians,^11^ although we did not find evidence describing the degree of prevalence or impact of broader policies to refuse service to Medicaid patients. It is not possible to definitively state from our analysis whether the high number of Hispanic and non-English speaking patients experiencing insurance transfers is causally attributable to a higher number of patients in these groups requiring care on Medicare and Medicaid plans compared to White, English-speaking patients. However, public data indicate that minoritized groups in the United States, including Hispanic and non-English-speaking patients, are more likely to require these safety net services for a complex set of reasons. Additionally, literacy in complex healthcare coverage requirements and benefits may be lower in these populations.^12^

The employer-based health insurance paradigm may also create disparities for minoritized individuals who face higher barriers to obtaining employment.^13^ The impact of this type of transfer on patient care has not been extensively researched. As discussed above, previous efforts to examine the impact of interfacility transfer in national datasets have found significant impacts, but these were not specific to patients transferred due to insurance status. Because of the requirements imposed by EMTALA, these patients should be medically stable before transport, and the clinical threshold for stability may be functionally higher given the reason for transfer is non-clinical. Further studies of the clinical impact of these transfers could add impact to research on their potential disparities.

Refusal of Medicaid services is likely due to the level of reimbursement provided by plans, and the same holds true for refusal of patients with Medicare Advantage plans. Medicare Advantage plans were developed as part of the Tax Equity and Fiscal Responsibility Act, in part as a mechanism to transfer risk from fee-for-service Medicare from the government to risk-based insurers who would cover members in exchange for a monthly per-capita payment.^14^ Payments for inpatient service from Medicare Advantage plans, however, may be 5.6% less than traditional fee-for-service Medicare.

An important balance exists for hospital administrators seeking to make insurance contracting decisions. On one hand, hospitals must make adequate financial recovery to continue serving patients and fulfilling their community mission. These decisions are even more challenging at academic hospitals as their mission includes education and research, both of which require substantial financial investment. Still, decisions not to accept Medicaid or other insurance plans can have important impacts on patients seeking care, and institutions must seriously consider the ethics of such practices, including, above all, a responsibility to not negatively impact patient care. Furthermore, our research suggests that impacts on patients may be disproportionately borne by minority groups. This adds another layer to the ethical implications of such practices and further entrenches systemic barriers to care for these patient populations. As discussed above, further research may help to elucidate the impact these transfers have on clinical care and assist hospitals in making decisions that appropriately balance reasonable reimbursement requirements with the duty to provide high-quality patient care.

### Previous Studies

Interfacility transfers have been studied in other, specific contexts such as in cancer patients^16^ and pediatric trauma patients.^17^ Studies specific to the ED include one that examined patients with ST-elevation myocardial infarction^18^ and one of patients experiencing inbound transfers to an academic ED.^19^ In each of these examples, insurance status was evaluated as a potential exposure to the overall risk of transfer, for any reason. In our study, we reviewed charts to determine the specific reason for transfer, such that insurance transfers could be explicitly reviewed. A benefit of this approach is that it removes potential confounders, including the possibility that socioeconomic variables impact the overall risk of transfer including for non-insurance reasons such as complexity of trauma. Previous studies have used national databases such as the National Inpatient Sample, which allows for greater generalizability although it may not permit the granularity of review included in our study.

## LIMITATIONS

This study represents a single-site review of trends in interfacility transfer for non-contracted insurance status. Previous studies have used national datasets to understand the impact of transfers on clinical care, although it is difficult to determine from these which transfers were due to insurance status. In our hospital’s records, while a code was applied to indicate the reason for transfer, it frequently read “Patient preference” in cases where non-contracted status was discussed in the medical record as the reason for that preference. This meant that manual review of transfers had to be performed to identify specific language in notes in the chart detailing insurance as the reason for transfer, and that wider study of this topic would likely require facility-by-facility review of datasets. Finally, our hospital ED is in an area where US Census data indicates a median income of $106,058. Therefore, our sample may not be reflective of the socioeconomic status of patients presenting to the ED in other locations.

## CONCLUSION

In our single-center ED study, minority patient populations were disproportionately impacted by interfacility transfers for non-contracted insurance status. We found increased transfer rates due to insurance incompatibility for all minority demographics queried. The most severe disparity was found for non-English speakers and patients of Hispanic ethnicity.

## Figures and Tables

**Table 1 t1-wjem-26-1696:** Percentage of demographic groups in cohort transferred from the emergency department (ED) due to non-contracted insurance vs patients admitted to the hospital directly from the ED.

	Number of insurance transfers	Total admitted	Total (admitted and transferred)	Percentage insurance transfer
Hispanic	49	3,686	3,735	1.31%
Non-White	45	4,004	4,049	1.11%
All non-White and White Hispanic	90	7,331	7,421	1.21%
White (non-Hispanic)	274	31,365	31.693	0.87%
Non-English speaking	24	1.142	1.166	2.06%
English speaking	342	37,791	38,133	0.90%
All minority groups combined*	100	7,617	7,717	1.30%
White, non-Hispanic, English-speaking	264	31,079	31,343	0.84%
Total transfers**	366	38,933	39,299	0.93%

* All minority groups combined” refers to patients who were non-White, Hispanic, or non-English speaking, adjusted for double-counting.

** Total transfers is not a sum of the columns above due to double-counting for patients who fit multiple subcategories.

**Table 2 t2-wjem-26-1696:** Odds ratio calculations for each minority group transferred from the emergency department due to non-contracted insurance status.

	Percentage insurance transfer	Comparison group	Odds ratio	95% CI	P-value
Hispanic ethnicity	1.31%	White, non-Hispanic	1.52	1.15–2.13	< .001**
Non-White race	1.11%	White, non-Hispanic	1.28	0.96–1.82	0.06
All non-White race and white Hispanic	1.21%	White, non-Hispanic	1.41	1.14–1.84	< .001**
White (non-hispanic)	0.87%	Reference	1.00	Reference	Reference
Non-English speaking	2.06%	English speaking	2.32	1.53–3.53	< .001**
English speaking	0.90%	Reference	1.00	Reference	Reference
All minority groups combined	1.30%	White, non-Hispanic, English speaking	1.55	1.23–1.95	< .001**
White, non- Hispanic, English-speaking	0.84%	Reference	1	Reference	Reference

** Comparison was made to reference groups as specified. Each reference group had a lower incidence of insurance transfers from the emergency department than any minority group analyzed.

**Table 3 t3-wjem-26-1696:** Number and percentage of patients with each insurance type transferred from the emergency department due to non-contracted insurance status.

Financial status	Number of transfers	Percentage of insurance transfers
Commercial Non-Contract	22	6.0%
Medicaid Non-Contract	149	40.7%
Medicare Advantage Non-Contract	194	53.0%
Self-Pay	1	0.3%
Total	366	100%
